# Racioethnic Disparities in Endometrial Cancer Outcomes

**DOI:** 10.3390/diagnostics14040417

**Published:** 2024-02-14

**Authors:** Ojone Illah, Deborah Adeeko, Adeola Olaitan, Aleksandra Gentry-Maharaj

**Affiliations:** 1 Department of Women’s Cancer, Elizabeth Garrett Anderson Institute for Women’s Health, University College London, London WC1E 6DD, UK; 2 Elizabeth Garrett Anderson Institute for Women’s Health, University College London, London WC1E 6AU, UK; 3 Medical Research Council Clinical Trials Unit, Institute of Clinical Trials and Methodology, University College London, London WC1V 6LJ, UK

**Keywords:** endometrial cancer, endometrial cancer disparities, racial disparities, black women

## Abstract

Black women are twice as likely to die from endometrial cancer (EC) compared with white women. This represents one of the worst racioethnic disparities amongst all cancers globally. Compared with white women, black women are more likely to be diagnosed with advanced EC, have more barriers to accessing care and experience increased delays in obtaining an EC diagnosis and commencing treatment. Histological and molecular differences place black women at higher risk of being diagnosed with more aggressive EC subtypes that carry less favourable outcomes. Furthermore, EC diagnostic pathways are less reliable in black women, and black women are less likely to receive evidence-based treatment for EC. This racioethnic disparity in EC outcomes exists both in the UK and US, despite differences in healthcare systems. This review methodically describes the key factors along the patient journey that contribute to the disparity in black women and proposes multifaceted approaches to lessen these gaps.

## 1. Introduction

Endometrial cancer (EC) is cancer of the lining of the uterus. It is the dominant form of uterine malignancy. In the UK and US, it is the fourth most common cancer affecting women and the most common gynaecological malignancy. In 2020, 11,385 women were diagnosed with EC in the UK, and 2613 women died from EC [1]. The global incidence of EC has steadily risen in the past few decades, and by 2040, mortality from EC is predicted to increase by 33% [2]. Obesity is a well-known risk factor for EC development. The rates of obesity have tripled in the last 50 years [3], and this increase in combination with longer life expectancy explains most of the rising incidence of EC.

Most ECs (70–80%) belong to the ‘low-risk’ group, which consists predominantly of endometrioid histotypes. Five-year survival in this group is favourable at 75–86%. The high-risk group comprises more aggressive histotypes, such as serous ECs with a five-year survival of 35% [4]. EC can often be diagnosed at an early stage, as many women will present early with abnormal vaginal bleeding. However, women diagnosed at an advanced stage and with high-risk histotypes have worse outcomes.

**Figure 1:**
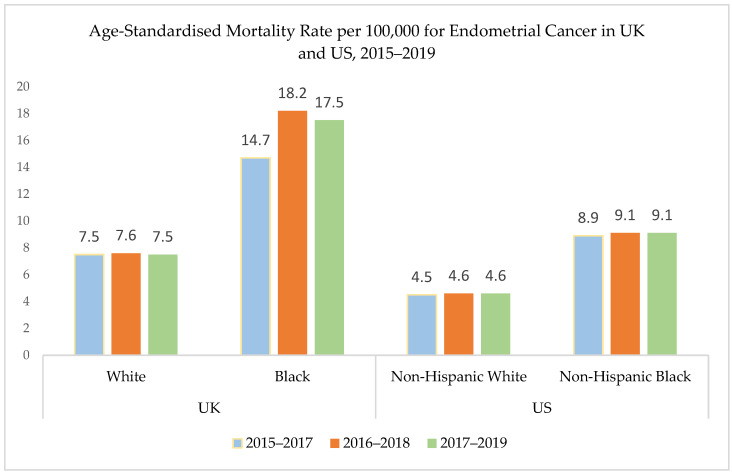
Differences in EC mortality in black and white women. Age-standardised mortality rate from EC in the UK and US between 2015 and 2019. Based on data from the UK Office of National Statistics (ONS) [7] and the US Statistics, Epidemiology, and End Results (SEER) Program [5]. Footnote: The ‘Black’ group from UK data refers to women from Black African, Black Caribbean, and Black Other ethnic groups.

In multi-ethnic populations such as the UK and the US, black women have a lower incidence of EC, but much higher mortality compared with white women. US data from the Surveillance, Epidemiology, and End Results (SEER) Program report a 9.1 per 100,000 EC mortality rate in black women, nearly double the mortality in white women (4.6 per 100,000) [5]. In the US, this results in a 21% lower five-year survival in black women compared with white women [6]. In the UK, mortality data from the Office of National Statistics (ONS) shows a similar disparity, with black women having significantly higher mortality from EC compared with other ethnic groups [7,8] (Figure 1).

This disparity in EC mortality is perhaps one of the worst racial disparities seen amongst all cancers. Other cancers that demonstrate similar racioethnic disparities in outcomes include prostate and breast cancer, which cause much higher mortality in black men and women compared with their white counterparts [9,10]. Several decades of research, predominantly from the US, has shown that black women are more likely to be diagnosed with high-risk EC histotypes. The incidence of these more aggressive EC subtypes has risen for all women, but the increase in black women has been twice as high [11]. Black women are also more likely to be diagnosed with EC at an advanced stage compared with white women [12]. Recent UK data show that African and Caribbean women are two times more likely to be diagnosed with advanced-stage EC compared with white British women [13], an association so strong that Cancer Research UK labelled ethnicity as a ‘significant factor’ in the stage at diagnosis of EC [13]. In this review, we will discuss the factors along the patient pathway that contribute to the racioethnic disparities seen in EC in multi-ethnic populations.

## 2. Risk Factors for Endometrial Cancer Development

Several modifiable and nonmodifiable risk factors are associated with EC development [14]. Inherited susceptibility to EC is seen most commonly in Lynch syndrome, where mutations in one of the four mismatch repair (MMR) genes result in a predisposition to EC [15]. This is not currently known to differentially affect racioethnic groups [16]. Age is another common nonmodifiable risk factor, as EC primarily affects postmenopausal women [14,15].

Many of the modifiable EC risk factors influence the balance of progesterone and oestrogen, which leads to changes in the endometrium that predispose women to EC. The endometrium undergoes repeated cycles of replication over the life course. EC is thought to develop from mutations that arise during these repeated replication cycles. Oestrogen is the primary hormone responsible for the proliferation of the endometrium, with progesterone conferring a protective effect. The balance of oestrogen and progesterone exposure is, therefore, an important determinant of EC development, where prolonged unopposed oestrogen exposure increases the risk of EC [17].

Obesity is strongly associated with the development of endometrioid EC [15]. The impact of obesity is two-fold, through the creation of a pro-inflammatory and hyper-oestrogenic state [15]. The rising incidence of EC across all ethnic groups can be partly explained by the parallel obesity epidemic. Obesity is associated with 40% of all EC cases in the UK and 57% of all EC cases in the US [18,19]. In these countries, black women have an increased prevalence of obesity compared with white women [20,21], and this has been suggested as a potential cause for the steeper rise in EC incidence and mortality in black women. However, most EC mortality in black women is caused by the more aggressive non-endometrioid EC subtypes not linked to obesity. The association between obesity and EC is the same in black and white women [22]. Thus, the increased obesity prevalence in black women cannot explain the racioethnic disparity in the stage at diagnosis and mortality.

Other risk factors for EC development include the use of exogenous unopposed oestrogen, the use of tamoxifen, physical inactivity, insulin resistance, and polycystic ovarian syndrome [14]. No racioethnic differences have been observed in the association between exogenous hormone use and EC development [22], or in the other known risk factors.

## 3. Disparities in the Pre-Diagnostic Patient Pathway

### 3.1. Access to Healthcare

Healthcare systems vary across the globe and, thus, disparities in access to care in EC vary geographically. At one end of the spectrum, the UK’s National Health Service (NHS) provides a free-at-point-of-need healthcare system, which is one of the highest ranked among high-income countries [23]. It may be reasonably expected that given the nature of the NHS, healthcare in the UK should be universally accessible and equitable. The healthcare system in the US, although very robust, requires patients to possess health insurance to receive treatment. In this setting, one can expect to see a much higher impact of socioeconomic factors on access to care. Despite these healthcare system differences, black women in both the UK and the US experience higher mortality from EC compared with white women. In environments where healthcare can be equally accessed, black women remain at risk of increased mortality. A US study based on data from the US Department of Defense’s Automated Central Tumor Registry demonstrated that black women remained at increased mortality risk despite controlling for healthcare access within the study population [24]. These findings suggest that differences in healthcare access only play a small role in the racioethnic disparity in EC mortality.

### 3.2. Patient-Related Delays

As there is no current screening test for EC, diagnosis is usually made after patients present with symptoms [25]. Postmenopausal bleeding (PMB) is the hallmark presentation of EC. Only 5–10% of women with PMB will be diagnosed with EC, although 90% of women diagnosed with EC will have had PMB [26]. Other clinical presentations are rarer and include abnormal vaginal bleeding in pre- and peri-menopausal women, and symptoms of locally advanced disease such as abdominal distention, pain, and bowel or bladder dysfunction [15].

**Figure 2:**
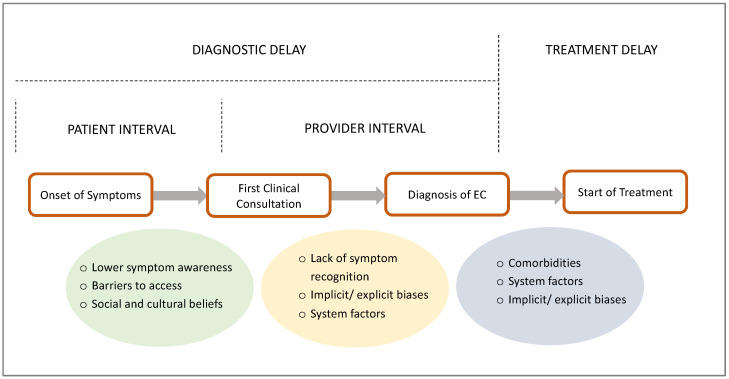
Summary of the patient diagnostic pathway. The patient pathway in EC leading to diagnosis and treatment, including types and causes of delays in the pathway.

EC detection first relies on the patient’s ability to recognise a symptom as abnormal and seek medical help. A prolonged interval between symptom development and medical review (patient interval) can result in diagnostic delays, potentially causing disease progression and more advanced disease at diagnosis. Shortening this interval is of utmost importance to achieving earlier diagnosis (Figure 2). In EC, low income, lower educational attainment and having no or publicly-funded insurance are factors that are known to contribute to patient-related delays [27,28,29]. Studies looking at racial differences in patient intervals relative to overall diagnostic delays in EC are sparse, and there are more data on other cancer types. An interview-based study of women diagnosed with breast cancer in the UK found that Black African women reported the longest interval between symptom onset and seeking medical help [30]. Another large UK cohort study of 126,627 participants found the diagnostic interval for several cancers to be seven days longer for black patients in the UK compared with white patients [31]. A 1996 study in the US found that the patient interval in EC did not differ significantly between black women and white women [32], but recent studies are lacking. In the literature, commonly reported reasons for patient delays include limited symptom awareness, barriers to accessing care, sociocultural and religious beliefs, and the use of alternative treatment [33,34].

Cancer symptom awareness is known to be lower in ethnic minority groups, which can lead to a prolonged patient interval and a consequent advanced disease stage at diagnosis [35,36]. A meta-analysis of 15 studies on cancer beliefs and literacy in multi-ethnic populations (UK, US, Canada, and Australia) confirmed a lower awareness of cancer causes and symptoms in ethnic minority groups [37]. No studies were found investigating EC symptom awareness in the UK, but a qualitative study of 15 black women diagnosed with EC in the US highlighted a lack of clarity around menopause and abnormal bleeding [38]. The predominant understanding of PMB in the study cohort was the resumption of ‘normal’ menstruation, and reporting to a medical doctor was influenced by an increased severity of bleeding or the onset of pain, both of which were correlated with significant delays [38].

Other barriers to symptomatic presentation in the UK in black women were explored in a survey of 720 women in England [33]. Black and other ethnic minority women had significantly more barriers to symptomatic presentation, including a lack of confidence in discussing symptoms with a general practitioner (GP), embarrassment in discussing gynaecological symptoms, and the use of traditional remedies [33]. Sociocultural beliefs play an important role in the health-seeking behaviours of individuals from black backgrounds. Fatalism is prevalent in many ethnic minority groups, wherein a diagnosis is seen as a ‘destiny’ and is predetermined [37]. Women from ethnic minority groups are more likely to pray about symptoms, further delaying clinical presentation [33,37]. A qualitative study of 937 Black African and Caribbean women in the UK found that attendance at religious services was associated with reduced compliance with cervical screening [39]. These patient delays are compounded by a mistrust of medical professionals by black patients [40].

In the US, the factors affecting symptomatic presentation may be confounded by differences in healthcare access and affordability. Studies, however, show similar findings in health-seeking behaviour among the black population. An examination of the medical records of EC cases in a single US centre found that black women had a significantly longer duration of symptoms prior to presentation compared with white women [41]. Another study in the US found that black women were less likely to be recognised as having PMB, both by patients and clinicians [42].

### 3.3. Care Provider Factors 

The primary physician plays an important role in the diagnosis of EC. Being the first port of call, they must be able to recognise symptoms as abnormal and refer to specialist services appropriately. In the UK, this role is fulfilled by the general practitioner (GP), who makes a referral using the suspected cancer two-week wait (2WW) pathway. The (care) provider interval refers to the period between initial primary care presentation and diagnosis [31], and shortening this interval shortens diagnostic delays and could improve cancer outcomes (Figure 2). Again, there are minimal data investigating EC diagnostic intervals in black women in the UK. Studies on other cancers show that racial inequalities in diagnostic intervals are inconsistent and not solely responsible for racioethnic disparities in cancer outcomes [31,43]. A study in the UK assessed racioethnic variations in the number of GP consultations with cancer symptoms prior to hospital referral. The study included multiple cancer sites, including EC. Compared with white patients, black patients had an increased probability of three or more GP consultations before hospital referral (odds ratio: 1.83, 1.51–2.23; *p* < 0.0001) [44]. This translates into black patients requiring more GP visits before specialist referral, perpetuating a provider-driven diagnostic delay. A single-centre study in the US found no significant differences in the time to specialist referral in EC; however, this was following diagnosis [41]. In a recent study on patients with EC in the US, black patients (n = 858) were more likely than white patients (n = 1749) to experience a delay in diagnosis after presenting with abnormal vaginal bleeding [45]. The same study found that black patients underwent more visits for the evaluation and management of abnormal vaginal bleeding before a diagnosis was made [45]. Most provider-related delays are often due to individual and systemic biases, which are difficult to assess scientifically and can only be inferred.

## 4. Disparities in Endometrial Cancer Diagnosis

**Figure 3:**
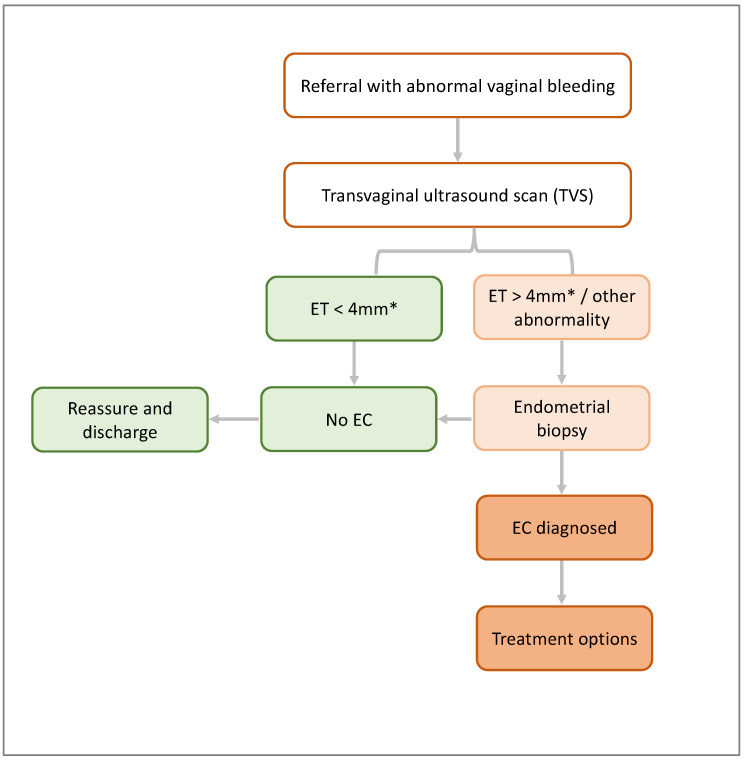
Summary of the EC diagnostic pathway. The diagnostic pathway for EC is similar in the UK and US, consisting of a TVS and endometrial biopsy dependent on TVS findings. TVS = transvaginal ultrasound, ET = endometrial thickness, and EC = endometrial cancer. Footnote: * Accepted as the norm but the cut-offs may vary according to the centre/country (3 mm, 4.5 mm, and 5 mm cut-offs are also used).

PMB is the most common symptom experienced by women with EC, and most will present to primary healthcare services. Between 5 and 10% of these women will have EC, but most will require speciality referral and additional investigations. In the UK, recommendations from the National Institute for Health and Care Excellence (NICE) guide EC referral and diagnostic pathways [46,47]. Transvaginal ultrasound (TVS) is the initial recommended investigation, during which the endometrial thickness and other features are assessed. Most centres in the UK employ a 4 mm cut-off for endometrial thickness, above which an endometrial biopsy is recommended [48]. Where the endometrial thickness is <4 mm but other endometrial abnormalities exist, such as an irregular endometrial lining or fluid in the cavity, a biopsy is also indicated. The guidance in the US from the American College of Obstetricians and Gynaecologists (ACOG) also recommends TVS as a first-line evaluation tool [49] (Figure 3).

TVS is non-invasive and can reliably exclude EC in most circumstances. However, in the presence of fibroids, clear views of the endometrium may be obscured, rendering TVS unreliable. Compared with white women, black women are three times more likely to have uterine fibroids [50]. Furthermore, TVS can be unreliable in the assessment of high-risk non-endometrioid endometrial tumours [51,52]. Endometrioid EC commonly presents with a thickened endometrium, which is visible on TVS. Other high-risk non-endometrioid EC histotypes do not reliably present with a thickened endometrium. In one study that retrospectively analysed the ultrasonographic findings in 52 patients with high-risk non-endometrioid EC histotypes, 17% had an endometrial thickness below 4 mm [53]. In another similar retrospective analysis of 58 patients with high-risk EC, 27.5% were recorded to have a thin (<5 mm) or indistinct endometrial lining [54]. A recent retrospective analysis of 139 serous EC cases similarly showed that nearly 25% of patients had an endometrial thickness below 4 mm [55]. Like fibroids, black women are more likely to be diagnosed with non-endometrioid EC subtypes [11]. Given the greater prevalence of fibroids and non-endometrioid EC in black women, the diagnostic accuracy of TVS in black women is questionable. Its use may potentially result in missed and delayed diagnoses.

These differences led Doll et al. in 2021 to conduct a retrospective study on a simulated cohort of 367,060 women with PMB [52]. Using an endometrial thickness cut-off of 4 mm, the sensitivity of TVS for EC detection in black women was 47.5% compared with 87.9% in white women [52]. Some of this difference was attributed to a higher prevalence of fibroids in black women. Where TVS views are obscured by fibroids in clinical practice, it is probable that alternate modes of imaging and/or endometrial biopsy will be sought. If views of the endometrium are clear and reported as normal in the presence of malignant pathology, this disparity is worrying.

Endometrial sampling is typically undertaken with Pipelle biopsy, dilatation and curettage, or hysteroscopic biopsy [56,57]. Pipelle biopsy and dilatation and curettage are blind sampling techniques, which can miss an EC diagnosis in up to 11% of cases when compared with hysteroscopic biopsy [56]. Different centres will have varying access to these sampling methods, and the gold-standard hysteroscopic biopsy may not be readily accessible. A study assessing for racioethnic differences in sampling techniques has not been found, making it unclear if this plays a significant role in EC racioethnic diagnostic disparities.

A recent study in the US found that 10.1% of black patients versus 5% of white patients diagnosed with EC did not receive a guideline-concordant diagnostic procedure [45]. Guideline-concordant procedures included endometrial biopsy, dilation and curettage, hysteroscopy, transvaginal or pelvic ultrasound, or pelvic magnetic resonance imaging. Instead, these patients were incidentally found to have EC during other investigations and/or procedures [45]. Where diagnostic procedures were performed, black patients compared with white patients were more likely to experience a delay in diagnosis, defined as >1 year following presentation with abnormal vaginal bleeding [45]. The authors proposed that barriers to accessing specialist cancer care may contribute to these reported diagnostic disparities [45]. 

Alternate diagnostic tests may be warranted to overcome the inconsistency of TVS. Recently, the use of DNA methylation testing for EC detection has been explored in various studies [58,59]. A recently published prospective cohort study in the UK compared the performance of a DNA-methylation-based test, the WID-qEC™ test, with ultrasonography for EC detection [59]. The WID-qEC™ test uses real-time polymerase chain reaction to detect methylation in two genes, *ZSCAN12* and *GYPC*, which are known to be associated with EC [59]. Conveniently, this test utilises non-invasive cervicovaginal swabs. In this study, 400 women with abnormal vaginal bleeding were assessed using the WID-qEC™ test as well as guideline-concordant ultrasonography. The results showed a superior diagnostic performance of the WID-qEC™ test compared with sonography, and the authors concluded that the use of the test in clinical practice may improve the diagnostic pathway [59]. The authors acknowledged the predominantly white study population as a limitation, highlighting that ongoing studies will assess the test’s performance in black women [59].

## 5. Histological and Molecular Differences

### 5.1. Histological Differences

Bokhman in 1983 classified EC into Type-I and Type-II tumours, based on clinical and hormonal features [60]. Type-I ECs are oestrogen-dependent and associated with obesity and hyperlipidaemia. These ECs have the best prognosis. Type-II ECs are oestrogen-independent, aggressive, and have a poorer prognosis [60]. More recent classification systems, however, incorporate the tumour morphology and genetic profile.

The World Health Organization (WHO) classifies EC by morphology into endometrioid carcinomas, serous carcinomas, clear-cell carcinomas, undifferentiated carcinomas, carcinosarcomas, and rarer subtypes, including neuroendocrine, mucinous, squamous-cell, and mesonephric carcinomas [61]. However, ECs are mainly grouped into endometrioid and non-endometrioid subtypes, and most non-endometrioid subtypes belong to the Type-II EC category [14]. Endometrioid carcinomas are the most commonly encountered, accounting for approximately 75% of all ECs [62]. These typically arise from a background of atypical endometrial hyperplasia and are graded based on architectural complexity into low-grade (grades 1–2) and high-grade (grade 3) types. Low-grade endometrioid ECs have the best survival outcomes [15]. High-grade endometrioid carcinomas and non-endometrioid carcinomas, notably serous carcinomas, are aggressive, with poor survival. Up to 50% of serous ECs have extrauterine disease spread at presentation compared with endometrioid EC, which typically presents at an early stage [15,63].

Histological differences are a dominant contributor to the racioethnic disparity in EC mortality. Black women are more likely to be diagnosed with higher-risk non-endometrioid EC subtypes [64,65]. Non-endometrioid subtypes are present in 28% of black women with EC compared with 10% in women of other ethnic groups [64]. One study analysed 248 EC samples from black and white women. The results showed a higher prevalence of serous EC (58% vs. 36%) and carcinosarcoma (25% vs. 16%), and a lower prevalence of endometrioid EC (17% vs. 48%) in black women [66]. A recent cohort analysis of the US SEER program concluded that EC grade and histological subtype explained 24.4% and 20.1% of the black–white EC mortality disparity, respectively [67]. In a separate analysis of the US National Cancer Database, the histologic subtype was deemed accountable for up to 56.2% of the excess relative risk among black patients with EC [68].

### 5.2. Molecular and Genetic Differences

**Figure 4:**
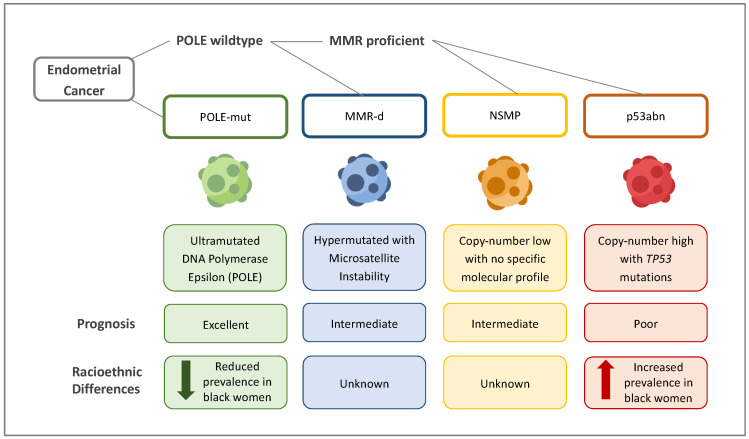
Molecular subtypes of endometrial cancer. The four molecular subtypes of endometrial cancer based on The Cancer Genome Atlas (TCGA) classification [69] and known racioethnic differences. POLE = DNA polymerase epsilon, MMR = mismatch repair genes, and NSMP = no specific molecular profile.

Within the last decade, The Cancer Genome Atlas (TCGA) has defined a molecular characterisation for EC based on copy number alterations and mutational burden [69]. This has given rise to four molecular subtypes, subsequently reclassified pragmatically for clinical use (Figure 4). As tumour aggressiveness is largely driven by molecular and genetic makeup, this molecular classification of EC combined with clinicopathological features offers a significant improvement in prognosis prediction and the possibility of tailoring treatment based on individualised stratification.

The four molecular groups are as follows (Figure 4) [15,69]:

(i) Ultramutated/DNA polymerase epsilon (*POLE*)-mutated (*POLE*-mut) ECs, characterised by mutations in the *POLE* region, which result in a high transversion mutation frequency. These affect younger women and have good clinical outcomes.
(ii) Hypermutated ECs with microsatellite instability (MSI)/mismatch-repair-deficient (MMRd) ECs, characterised by a ten-fold higher mutation frequency compared with MMR-proficient tumours. Ten per cent of these mutations are due to germline defects in the MMR gene (Lynch syndrome). The remainder are due to somatic defects. ECs in this group have an intermediate prognosis.
(iii) Copy-number-high/p53-abnormal (p53abn) ECs, characterised by mutations in *TP53*. Most serous ECs and carcinosarcomas belong to this group, which is associated with the worst clinical outcomes.
(iv) Copy-number-low/no specific molecular profile group (NSMP) ECs, which largely consist of endometrioid ECs and have stage-dependent intermediate–excellent clinical outcomes.

The p53abn molecular group is associated with the most aggressive EC subtypes. Despite being responsible for only 15% of EC cases, it is responsible for 50–70% of EC mortality [70]. *POLE*-mutated tumours, contrarily, have the most favourable survival outcomes [70]. Inactivation of the *PTEN* tumour suppressor gene is frequently associated with endometrioid ECs. In EC, *PTEN* mutation is associated with favourable outcomes [71]. Black women with EC are three times more likely to have mutations in *TP53* [72,73] but less likely to have the more favourable *POLE* and *PTEN* mutations [74]. A recent study sequenced 1882 ECs and found that ultramutated *POLE*, associated with the best outcomes, is rare in black women [75]. Mutations in proto-oncogene HER2/neu are also more frequent in black women [12]. This mutation is associated with serous EC and worse clinical outcomes. ECs that test positive for HER2/neu mutations may benefit from treatment with the Her2/neu monoclonal antibody trastuzumab [70].

Most EC cases are caused by sporadic mutations such as those described above. Approximately 5% of ECs are caused by inherited mutations, such as mutations in MMR genes in Lynch syndrome and mutations in *PTEN* in Cowden syndrome [14,15]. Little is known regarding the prevalence of MMR deficiency in women from different racioethnic backgrounds. A small study based at the University of Miami found that MMR deficiency in EC was detected evenly across all racioethnic groups [16]. In other cancers that feature MMR deficiency, such as colorectal cancers, there are no reported differences in MMR deficiency between different racioethnic groups [76].

Additionally, unknown molecular and genetic markers may further explain the racioethnic EC mortality disparity. Unfortunately, the TCGA discovery analysis that led to the new molecular classification system did not include a significant number of specimens from black participants with EC [77]. A small number of studies have aimed to profile genomic and proteomic markers associated with EC in black women [74,77]. These studies have discovered several biomarkers, but much larger studies are required to validate these findings.

### 5.3. Updated FIGO Staging

Cancer staging is intended to inform prognosis, with advanced disease stages being indicative of a worse prognosis. Traditionally, EC staging has relied on the anatomical extent of the tumour based on clinical, radiological, and pathological parameters. It is now acknowledged that EC is a heterogeneous disease with significant variations in outcomes, which additionally depend on histological and molecular EC subtypes. Upon review of the available evidence, the International Federation of Gynaecology and Obstetrics (FIGO) in June 2023 introduced an updated EC staging system (Table 1) that incorporates histological and molecular classifications of EC [78]. 

With the new staging system, the presence of aggressive EC histotypes results in an advanced EC stage. Aggressive/high-grade EC histotypes are serous adenocarcinomas, clear-cell adenocarcinomas, mesonephric-like carcinomas, gastrointestinal-type mucinous endometrial carcinoma, undifferentiated carcinomas, carcinosarcomas, and grade-3 endometroid adenocarcinomas [78]. In the presence of these histotypes, even where the disease is confined to the endometrium or a polyp, a minimum of stage 1C disease is assigned [78]. 

The FIGO 2023 staging system encourages the performance of EC molecular subtyping for prognostic risk stratification and to inform treatment decisions [78]. According to the new system (Table 1), *POLE*mut is associated with a good prognosis, MMRd and NSMP with an intermediate prognosis, and p53abn with a poor prognosis [78]. In stage 1 and 2 EC, *POLE* and p53 mutations modify the stages as follows: in the presence of a *POLE*mut or p53abn EC—regardless of the degree of myometrial invasion, cervical involvement, the degree of lymphovascular space invasion (LVSI), and the histological type—the allocated disease stage will be 1A and 2C, respectively, with the molecular classification included as a subscript (Table 1) [78]. With these changes to the staging system, it is probable that the already existent racioethnic difference in the stage at EC diagnosis will increase.

## 6. Disparities in Endometrial Cancer Treatment

EC treatment consists of surgery, radiotherapy, and chemotherapy. The treatment choice depends on the EC stage, grade, and patient factors. However, the mainstay of EC treatment is surgery by hysterectomy, bilateral salpingo-oopherectomy, and surgical staging, followed by adjuvant therapy if indicated based on the pathology results. Recently, immunotherapy by means of immune checkpoint inhibition has been introduced for the management of advanced or recurrent EC [79]. Response to immunotherapy varies based on the molecular EC subtype [79].

### 6.1. Evidence-Based Care

Research into disparities in EC treatment has almost exclusively been conducted in the US, where Huang et al., in 2020, published five quality metrics for EC treatment based on the current guidelines and literature. These metrics are (i) surgical treatment within six weeks of diagnosis, (ii) use of a minimally invasive surgical approach, (iii) pelvic nodal assessment, (iv) adjuvant radiation, and (v) systemic chemotherapy. These quality metrics were evaluated in a retrospective review of EC cases from the US National Cancer Database. Authors found that black women were significantly less likely to receive care adherent to four out of five of these metrics compared with white women. Overall, white women received ‘perfectly’ adherent care 53.1% of the time, compared with 41.7% of black women [80]. Where care for black women was ‘perfectly’ adherent to guidelines, outcomes were improved, but there remained an increased mortality risk in black women [80]. This further demonstrates the multifactorial causality of disparate outcomes of EC in black women. Other studies compared the US SEER and National Cancer Databases against the standard of care as set out by the National Comprehensive Cancer Network [81,82]. These both showed that black women were less likely to receive guideline-concordant care, which was associated with worse overall survival.

The reasons behind the substandard care black women receive align with unequal access to healthcare and resources. In the US, the quality of care received is closely linked to geographical location, proximity to specialist services [81], and the type of insurance held. However, it is evident that these disparities persist even when access to care is free at the point of delivery, as in the UK. The role of discrimination and biases within healthcare systems and healthcare professionals, therefore, cannot be overlooked [81]. 

### 6.2. Delays in Treatment

Several studies in the US have shown that black women experience increased delays in the receipt of treatment following EC diagnosis. One study retrospectively analysed delays in treatment for all gynaecological malignancies between 2004 and 2017. A subgroup analysis of 406,044 EC cases identified a 4.2-day delay in treatment in black women compared with white women. Authors concluded that this delay resulted in a relative 25% increased mortality risk [83]. Another study prospectively assessed the time taken to start treatment for EC in 284,499 patients. The time taken to start EC treatment in African American women was 4–5 days more compared with women of other ethnicities [84]. In the US, these delays may also be related to insurance status and the type of treatment facility. Delays in insurance approvals can often influence the timing of the receipt of treatment [84].

### 6.3. Surgical Management

Research into disparities in surgical management of EC in the US has found that black women are less likely to receive surgery for EC and more likely to receive upfront radiotherapy, even in the presence of local disease only [85,86]. In a recent study of 23,431 women with low-risk stage-1A EC from the US SEER and National Cancer Databases, black women were significantly less likely to undergo hysterectomy compared with white women [87]. Among black women, 13.5% refused surgery compared with 8% of white women, but this difference was not significant. Rates of radiation and chemotherapy treatment were significantly higher in black women [87]. This difference in the proportion of women undergoing surgery as their first-line treatment has also been found across all grades of EC [88,89]. The proposed reasons for this disparity include differences in access to care, implicit biases, differences in rates of treatment refusal, and differences in co-morbidities [12,87].

Minimal access surgery for EC is possible by laparoscopic or robotic-assisted approaches. These are associated with fewer post-operative complications compared with open approaches [90]. Studies show that black women in the US are significantly less likely to undergo minimal-access hysterectomies compared with white women for benign gynaecological conditions [91]. For EC management, black women are also less likely to undergo minimal-access surgery compared with white women [92,93]. In the UK, an analysis of Hospital Episode Statistics data in England was conducted by Moss et al. in 2020. This found that black women had significantly lower rates of minimal-access surgery for EC (40.4%) compared with white women (58.6%) and Asian women (56%) [92]. In the US, a retrospective analysis of the American College of Surgeons’ National Surgical Quality Improvement Project’s database similarly found that black women were less likely to undergo minimal-access surgery for EC (49.3%) compared with white women (71.3%) [93]. This disparity may be related to geographical differences in access to specialist services and may be alleviated if patients undergo surgery at hospitals that perform more minimal access procedures [92]. 

Following surgery, black women also have a higher incidence of post-operative complications compared with white women [93,94,95]. A study in the US reported a 22.5% 30-day complication rate in black women compared with 13.6% in white women. When the complication rate was compared for minimal-access surgery alone, no difference was noted between the two groups [93], suggesting that this disparity may be mitigated by equitably performing minimal-access surgery. The higher readmission rate following surgery for EC in black women may also be mitigated by the optimisation of preoperative care [94].

### 6.4. Treatment Refusal

Treatment refusal may explain some of the disparity observed in the receipt of guideline-concordant care for EC. For many cancer types, black patients are more likely to refuse recommended treatment compared with other ethnic groups [96,97,98]. Significantly higher rates of treatment refusal have been described in EC for surgery [99] and chemotherapy [96], but not for radiotherapy [96,100]. Despite the significant racioethnic difference in the acceptance of chemotherapy treatment, a study found that this only adds a survival advantage of approximately two months to black women [96]. Authors used a causal mediation analysis to estimate the degree to which chemotherapy refusal impacted overall survival in black women and found this to be minimal (4.9%) [96]. They concluded that the impact of treatment refusal is minor and can only explain a small part of the differences in mortality and survival between black and white women.

## 7. Gaps in Knowledge and Potential Solutions

Addressing the racioethnic disparities in EC outcomes requires a multifaceted approach (Table 2). In the UK, national initiatives to address racial inequalities in women’s health have been established by the Royal College of Obstetricians and Gynaecologists [101]. It is clear that increased public awareness of EC and its symptoms is key to addressing patient factors that contribute to delays in EC diagnosis. The ‘You need to know’ campaign, launched in 2023, is a collaborative effort between the North East London Cancer Alliance and The Eve Appeal charity. The initiative aims to increase awareness of EC amongst black and Asian ethnic groups [102]. Expansion of this campaign and the development of similar targeted initiatives can enhance symptom awareness in at-risk communities. To ensure the success of these initiatives, messages delivered must be culturally sensitive, and individuals involved must be familiar with the unique experiences, beliefs, and values present in different ethnic groups. 

Globally, there is a need for more biological research into EC in women of different ethnicities and ancestral backgrounds, understanding that while race is helpful in defining disparities, it is a social and not a biological construct. Women from black ethnic backgrounds have been underrepresented in most cancer research to date [103,104]. This may inadvertently preclude them from benefitting from important developments in cancer research, particularly in the fields of cancer metabolomics and precision oncology. Ensuring racioethnic diversity in research is important to ensure that future developments are beneficial and applicable to patients of all racioethnic groups. Based on research findings, the development of race-adjusted guidelines and treatments may help improve outcomes in black women. An example of this is the GUIDE-EC project currently ongoing in the US which aims to ensure that guidelines are adjusted to ensure TVS performs better in the EC diagnostic pathway in black women [105].

## 8. Conclusions

Racioethnic disparities in EC outcomes are significant, with black women having considerably higher mortality from EC compared with white women. These disparities have been reported for several decades in the US literature and are supported by recently published UK data. The novel aspect of this review is its incorporation of both US and UK data to explore the impact of healthcare systems on these disparities. In examining the reasons behind these disparities, multiple contributory factors have been highlighted. Black women are more likely to be diagnosed with advanced-stage EC due to a combination of social and biological factors. Low symptom awareness and sociocultural beliefs that hinder health-seeking cause delays in clinical presentation. Care provider biases are assumed to cause further delays in diagnosis, and current EC diagnostic methods may miss EC diagnoses more frequently in black women. Black women are also more likely to be diagnosed with aggressive EC histotypes and have molecular tumour alterations associated with worse clinical outcomes. Finally, black women are less likely to receive guideline-concordant treatment for EC. With the exception of histological and molecular differences in EC in black women, the remaining factors responsible for the disparity are modifiable with appropriate interventions.

To begin to address the EC disparity, equity in healthcare is needed. At the population level, this involves increasing EC awareness across all racioethnic groups using culturally sensitive methods. In clinical settings, the implementation of mandates to standardise diagnostic and treatment pathways will help minimise the impact of individual and structural biases. Healthcare resources should be equitably redistributed to meet the needs of black women who experience worse outcomes from EC. This requires acceptance and support from decision-makers and funding streams. Importantly, there is a need for more scientific research which investigates and addresses the root causes of the EC racioethnic disparity. This would require incorporating measures to ensure increased research participation of black individuals who are under-represented in most cancer research. These factors that are modifiable should be prioritised in multi-ethnic populations such as the UK and the US, where equitable access to quality, evidence-based medicine can be expected by all, regardless of race or ethnicity.

## Figures and Tables

**Table 1:** FIGO 2023 staging of the endometrium. Adapted from Berek et al. [78]. See Section 5.3 for a detailed explanation. LVSI = lymphovascular space invasion, POLEmut = DNA polymerase epsilon mutated, and p53abn = p53 abnormal.

Stage	Description	
Stage 1	Confined to the uterine corpus and ovary	
	1A	Disease limited to the endometrium OR non-aggressive histological type, i.e., low-grade endometroid, with invasion of less than half of myometrium with no or focal LVSI OR good prognosis disease. 1A1: Non-aggressive histological type limited to an endometrial polyp OR confined to the endometrium. 1A2: Non-aggressive histological types involving less than half of the myometrium with no or focal LVSI. 1A3: Low-grade endometrioid carcinomas limited to the uterus and ovary.
	1B	Non-aggressive histological types with invasion of half or more of the myometrium, and with no or focal LVSI
	1C	Aggressive histological types limited to a polyp or confined to the endometrium
Stage 2	Invasion of cervical stroma without extrauterine extension OR with substantial LVSI OR aggressive histological types with myometrial invasion	
	2A	Invasion of the cervical stroma of non-aggressive histological types
	2B	Substantial LVSI of non-aggressive histological types
	2C	Aggressive histological types with any myometrial involvement
Stage 3	Local and/or regional spread of the tumour of any histological subtype	
	3A	Invasion of uterine serosa, adnexa, or both by direct extension or metastasis 3A1 Spread to ovary or fallopian tube (except when meeting stage 1A3 criteria) 3A2 Involvement of uterine subserosa or spread through the uterine serosa
	3B	Metastasis or direct spread to the vagina and/or to the parametria or pelvic peritoneum 3B1 Metastasis or direct spread to the vagina and/or the parametria 3B2 Metastasis to the pelvic peritoneum
	3C	Metastasis to the pelvic or para-aortic lymph nodes or both 3C1 Metastasis to the pelvic lymph nodes (3C1 i—micrometastasis; 3C1 ii—macrometastasis) 3C2 Metastasis to para-aortic lymph nodes up to the renal vessels, with or without metastasis to the pelvic lymph nodes (3C2 i—micrometastasis; 3C2 ii—macrometastasis)
Stage 4	Spread to the bladder mucosa and/or intestinal mucosa and/or distance metastasis	
	4A	Invasion of the bladder mucosa and/or the intestinal/bowel mucosa
	4B	Abdominal peritoneal metastasis beyond the pelvis
	4C	Distant metastasis, including metastasis to any extra-or intra-abdominal lymph nodes above the renal vessels, lungs, liver, brain, or bone
Stage	Molecular findings in patients with early endometrial cancer (Stages I and II after surgical staging)	
Stage 1Am_POLEm_	*POLE*mut endometrial carcinoma, confined to the uterine corpus or with cervical extension, regardless of the degree of LVSI or histological type	
Stage 2Cm_p53abn_	p53abn endometrial carcinoma, confined to the uterine corpus with any myometrial invasion, with or without cervical invasion, and regardless of the degree of LVSI or histological type	

**Table 2:** Summary of the racioethnic disparities in EC and proposed solutions. EC = endometrial cancer; TVS = transvaginal ultrasound.

Disparity	Summary	Proposed Solutions
Access to care	Socioeconomic differences between racioethnic groups are commonly attributed to disparities in outcomes. However, studies in equal-access healthcare systems demonstrate black women still have higher EC mortality.	Redistribution of resources to ensure equitable healthcare access.
Patient-related diagnostic delays	The following patient factors are more prevalent in black women and are associated with delays in clinical presentation: Lower cancer symptom awareness and health literacy; Social and cultural beliefs; Religious beliefs and fatalism; Mistrust of medical professionals.	Increased population EC awareness through culturally sensitive campaigns.
Care-provider-related diagnostic delays	There are minimal data on EC-specific care-provider-related diagnostic delays; studies on other cancers show black patients require more consultations with primary care providers before specialist referral. Inferred role of individual biases.	Reinforcement of mandates that standardise clinical pathways to limit the impact of individual biases.
Disparities in EC diagnostic pathway	EC diagnosis is largely reliant on TVS detection of endometrial abnormalities. In black women, TVS is less reliable due to the following: An increased prevalence of fibroids, which distort the endometrium; An increased prevalence of non-endometrioid EC histotypes, which can have a normal TVS appearance.	Review of thresholds for endometrial sampling and the development of diagnostic tests with improved performance.
Histological and molecular differences	Black women are more likely to be diagnosed with more aggressive EC due to the following: ■ An increased incidence of non-endometrioid histological EC types, e.g., serous EC and carcinosarcoma; ■ A higher prevalence of molecular alterations associated with poor outcomes (e.g., *TP53* mutations) and lower prevalence of favourable molecular alterations (e.g., POLE and PTEN mutations).	Histological and molecular differences are biological and nonmodifiable. More scientific research into non-endometrioid ECs may generate more effective treatment options.
Disparities in EC treatment	Several disparities in EC treatment in black women have been identified: Black women are less likely to receive guideline-concordant EC care and experience more delays in onset of treatment; Black women undergo surgery for EC less frequently. Where surgery is performed, they are less like to have minimal-access surgery and more likely to have surgical complications; Black women are more likely to refuse EC treatment	Reinforcement of mandates that standardise clinical pathways to limit the impact of individual biases

## Data Availability

Data sharing is not applicable.
